# Productivity losses attributable to headache, and their attempted recovery, in a heavy-manufacturing workforce in Turkey: implications for employers and politicians

**DOI:** 10.1186/s10194-015-0579-4

**Published:** 2015-11-14

**Authors:** H. Macit Selekler, Gürsel Gökmen, T. Müge Alvur, Timothy J. Steiner

**Affiliations:** Department of Neurology, Kocaeli University Medical Faculty, İzmit, Kocaeli Turkey; Company Health Services, Ford Otomotiv Sanayi AŞ, Gölcük, Kocaeli Turkey; Department of Family Medicine, Kocaeli University Medical Faculty, İzmit, Kocaeli Turkey; Department of Neuroscience, Norwegian University of Science and Technology, Trondheim, Norway; Division of Brain Sciences, Imperial College London, London, UK

**Keywords:** Headache disorders, Burden, Cost, Lost productivity, Work impact, Health care, Global campaign against headache

## Abstract

**Background:**

Headache disorders cause substantial productivity losses through absenteeism and impaired effectiveness at work (presenteeism). We had previously found these losses to be high in a mostly male, heavy-manufacturing workforce at Ford Otomotiv Sanayi AŞ (FO), in north-western Turkey. Here we aimed to confirm this finding in a year-long study to eliminate any effect of seasonal variation. The question then was how much of this lost productivity could be recovered by the effective provision of headache care.

**Methods:**

We used the HALT-30 Index to estimate productivity losses, surveying FO’s entire workforce (*N* = 7,200) during annual health-checks provided by the company’s on-site health clinic. Then we established, and widely advertised, a headache clinic within the same health clinic, providing specialist care free for 15 months. Outcome measures were HALT-30, company sickness records and the HURT questionnaire.

**Results:**

Usable data were collected from 5,916 employees (82.2 %; 5,485 males [92.7 %], 431 females [7.3 %]; mean age 32.5 ± 5.4 years). One-month headache prevalence was 45.4 % (*n* = 2,688). Productivity losses were reported by 968 employees (16.4 %) and, per affected employee, increased from 0.23 to 7.56 days/month as headache frequency increased (*P* <0005). Employees reporting headache on ≥15 days/month (*n* = 64; 1.1 %) accounted for 21.1 % of productivity losses, those with headache on 10–14 days (*n* = 104; 1.8 %) another 18.5 %. With increasing headache frequency, absenteeism/presenteeism ratio (overall 1:16) declined from about 1:4 to about 1:25 in those with headache on ≥10 days/month. Headache frequency and lost productivity were higher in females than males (*P* <0.0005). Both absenteeism and presenteeism rates declined after age 34 years (*P* <0.0005).

Only 344 employees with headache (12.8 %) requested appointments, and only 211 (7.8 %) actually consulted. Attendance was related to headache frequency (*P* <0.0005). Too few returned for follow-up to allow useful outcome assessment.

**Conclusion:**

The high productivity losses in this young mostly male workforce correlated with but were not wholly explained by headache frequency. A small minority of employees with high-frequency headache contributed highly disproportionately to the productivity losses. These should be the target of interventions aimed at productivity recovery. It is not clear what form such interventions should take: making headache care optimally available is not of itself sufficient.

## Background

Headache disorders are now clearly recognised among the major causes of disability within populations [1]. Impaired productivity and reduced output are measurable consequences at national level [2–6], and the financial costs are high [7, 8].

In our earlier report of productivity losses attributed to headache disorders at Ford Otomotiv Sanayi AŞ (FO), a vehicle-manufacturing company in north-western Turkey, we described losses through absenteeism and, much more substantial, those through impaired effectiveness at work (presenteeism) [9]. We estimated that 2.4 % of workforce capacity was surrendered to headache, 94 % of this due to presenteeism. Such high productivity losses in a largely male workforce might be surprising, but possible factors were the nature of the work—manual labour for two thirds, often heavy—and the recurring schedule disturbances of shift-work. We noted that a small minority (5.7 %) of those with headache, who were only 2.5 % of the workforce, accounted for >45 % of presenteeism-related lost productivity.

Here we report a continuation of the original observational study, and a follow-up interventional study in the same factory and workforce. We recorded productivity losses within the entire workforce over a period of one year, which allowed for any seasonal variation. Then, in the interventional study, we established a headache clinic within the factory’s on-site health clinic providing specialist ambulatory care free to employees. The purpose was to remove barriers to care in order to investigate the extent to which lost productivity attributable to headache could be recovered by the effective provision of care.

The study was conducted as a project within the Global Campaign against Headache [10–12].

## Methods

### Ethics

Since this was a project in preparation for and providing a clinical service, it fell outside the scope of research ethics review. While it involved access to company absenteeism records, these were held legitimately by the employer.

Data-protection legislation was complied with. No personal information derived for the project passed beyond the confines of the health clinic without first being made anonymous.

### Setting and population

At its manufacturing site in Gölcük, FO has a workforce of 7,200 employees: about two thirds are manual workers (90 % male) who rotate weekly through early, late and night shifts, each of 8 h; the remainder are clerical or managerial staff working a standard 5-day week, 8 am to 6 pm.

FO maintains sickness-absence records for its employees, which do not, however, include reasons for absence. The company provides an on-site health clinic, staffed *inter alia* by primary health-care physicians and nurses and providing care for day-to-day ailments. As a service to its employees, the clinic also carries out and records annual health-checks. These are offered, on a rotating basis, to about 10 % of the workforce each month.

### Lost productivity assessment

We used the Headache-Attributed Lost Time (HALT) Index [13] translated into Turkish as the assessment instrument, as in our earlier study [9]. HALT enquires into lost productivity during a preceding period: its first two questions ask about absenteeism due to headache and reduced productivity whilst at work with headache (presenteeism). To estimate total productive time lost per employee, reported in days/month, we added days wholly lost through absenteeism and days of presenteeism with <50 % productivity; by way of counterbalance, we ignored headache-affected days in which productivity was nevertheless >50 %. We adopted the HALT-30 version, which effectively sampled each individual’s headache pattern over the preceding 30 days. We assumed, in the total population, annual lost time was correctly estimated from the sum of individual HALT-30 scores x 12. For purposes of comparison, we also calculated work loss per week due either to absenteeism or to presenteeism. We took one work day as 8 h, and 1 month as 4 weeks, so that:

$$ \left[\mathrm{lost}\ \mathrm{days}/\mathrm{month}\ast 8\right]/4=\mathrm{lost}\ \mathrm{hours}/\mathrm{week} $$

Over a 1-year period, HALT-30 was administered to every employee attending his or her annual health-check, along with the clinic’s own materials related to the health-check. These forms were completed prior to the health-check and handed to the supervising physician.

### Data entry and management

Questionnaires were collected day by day and the data transferred to Excel by one health technician. Subsequently, all Excel sheets were converted to SPSS (Statistical Package for Social Sciences for Windows version 15.0).

### Statistics

We used SPSS for descriptive statistical analyses, calculating (as appropriate) means, standard deviations and medians. We used Student’s t-test, Pearson Χ^2^ and Mann Whitney tests for significance of differences, and Spearman or Pearson correlation coefficients for associations. We regarded *P* <0.05 as significant.

### Intervention

With full support from senior management, we established the headache clinic within the factory’s on-site health clinic, with one of us (HMS), a headache specialist, providing ambulatory services on 2 days per week. The service was advertised through posters at 28 sites in the factory, through the factory’s intranet, by an interview with HMS published in the factory’s monthly magazine and by an educational talk about headache disorders and the headache clinic given by HMS in the factory conference hall and open to all employees. Appointments could be made by any employee, without formality, through the secretary of the health clinic. There was no selection or prioritisation according to HALT-30 findings from the lost-productivity assessment: a request for an appointment from any employee was sufficient.

The headache service provided information, education and life-style advice as part of good management, and applied European principles of management [14] in allocating time to new and follow-up appointments, in use of ICHD diagnostic criteria [15], in selection of first-, second- and third-line drugs and doses, in use of prophylactics and in follow-up policy. Full records were kept. Although the service itself was free to employees, generally there were mandatory contributions of 20 % to the costs of prescribed medications under the Turkish health-care system.

The intervention continued for 15 months.

### Outcome assessment

The principal measure of outcome was estimated days of lost work-productivity per employee per year. We planned three semi-independent approaches in order to assess the effect of intervention.

#### HALT-30 in treated employees

HALT-30 was completed by patients when first attending for headache treatment, and again on discharge. The change would be the primary outcome measure.

#### HALT-30 in the entire workforce

We continued to administer HALT-30 in the annual health checks of all employees throughout the intervention study. This method would provide data with a response rate of close to 100 %, and include the effect of headache in people who did not seek health care for it (who might be the majority of those affected).

#### Company sickness records

There were complete records of sickness absences for the entire workforce, although no reasons were captured. We would calculate the total absences (days/week) for the workforce throughout the period of the study. In a secondary analysis, we would look only at absences of ≤5 days’ duration (those of longer duration were less likely to be headache-related). We did not intend to examine individual sickness records, since this might be objectionable to participating employees and might influence their behaviour.

### Clinical outcome

We assessed clinical outcomes in each treated employee during follow-up and on final discharge using the HURT questionnaire [16].

## Results

### Observational study

Of the 7,200 total workforce, HALT-30 data were collected from 5,940 (participation rate 82.5 %). Usable data were available from 5,916: those with scores higher than 60 (the maximum possible) were discarded, as were those who reported productivity loss (presumably from other causes) but not headache. This sample (82.2 % of the workforce) consisted of 5,485 males (92.7 %) and 431 females (7.3 %) with a mean age of 32.5 ± 5.4 (range 20–57) years; only 550 (9.3 %) were aged ≥40 years.

The 1-month headache prevalence was 45.4 % (*n* = 2,688). Productivity loss was reported by 968 employees, who were 16.4 % of the sample and 36.0 % of those with headache, and totalled 6,452 hours/week (380 hours/week due to absenteeism and 6,072 hours/week due to presenteeism). Mean lost productive time per employee reporting any productivity loss (absenteeism and/or presenteeism) was 6.7 hours/week. We divided the headache-affected group into five sub-groups according to headache frequency (Table 1). Over half (1,385; 23.4 % of the entire sample) reported 2–4 attacks, while 90 (1.5 %) reported headache on ≥15 of the preceding 30 days. We observed a steep increase in productivity loss per employee, from 0.23 to 7.56 days/month, as headache frequency increased (Table 1) (Spearman correlation for absenteeism: 0.543 [*P* <0.0005]; for presenteeism: 0.149 [*P* <0.0005]). As a consequence, the small sub-group reporting headache on ≥15 days in the preceding month (*n* = 64), who were 1.1 % of the entire sample, accounted for 21.1 % of all productivity losses, and the only moderately larger sub-group (*n* = 104; 1.8 % of the sample) reporting headache on 10–14 days were responsible for another 18.5 % (Table 1).

**Table 1:** Absenteeism, presenteeism, total productivity losses and average productivity losses per employee with headache according to headache frequency

Headache frequency (days/month)	N (% of sample)	n (% of sample) [% of N] with productivity loss	Absenteeism (total days in preceding 30 days)	Presenteeism (total days in preceding 30 days)	Ratio absenteeism to presenteeism	Total productivity loss (total [%] days in preceding 30 days)	Average productivity loss per employee (days/month)
≤1	627 (10.6)	120 (2.0) [19.1]	30	112	0.27	142 [14.7]	0.23
2–4	1,385 (23.4)	442 (7.4) [31.9]	66	857	0.08	923 [28.6]	0.67
5–9	438 (7.4)	238 (4.0) [54.3]	47	806	0.06	853 [26.4]	1.95
10–14	148 (2.5)	104 (1.8) [70.3]	21	577	0.04	598 [18.5]	4.04
≥15	90 (1.5)	64 (1.1) [71.1]	26	654	0.04	680 [21.1]	7.56
Totals	2,688 (45.4)	968 (16.3) [36.0]	190	3,036	0.06	3,226 [100]	1.20

We also observed a consistent decline in absenteeism/presenteeism ratio with increasing headache frequency, from about 1:4 in those reporting one headache in the preceding month to about 1:25 in those with headache on ≥10 days/month (Table 1).

We investigated the effects of gender, age and nature of work on absenteeism and presenteeism. Mean headache attack incidence per month was higher in females (2.9 ± 4.3) than in males (1.6 ± 3.0; *P* <0.0005 [Student’s t-test]). Mean rates of absenteeism per employee were 0.03 ± 0.32 (median 0) days/month in females and 0.03 ± 0.39 (median 0) in males (*P* = 0.404 [Mann-Whitney]) and of presenteeism were 1.16 ± 2.51 (median 0) for females and 0.46 ± 1.67 (median 0) for males (*P* <0.0005 [Mann-Whitney]).

Both absenteeism and presenteeism rates were age-related (Table 2). After age 34 years, mean absenteeism per employee fell from 0.04 ± 0.45 to 0.01 ± 0.15 days/month (*P* <0.0005 [Student’s t-test]) and mean presenteeism from 0.58 ± 1.91 to 0.36 ± 1.37 days/month (*P* <0.0005 [Student’s t-test]).

**Table 2:** Absenteeism, presenteeism and total productivity loss (mean days/month) per employee according to age

Age range (years)	20–24	25–29	30–34	35–39	≥40
	(*n* = 415)	(*n* = 1,454)	(*n* = 2,161)	(*n* = 1,331)	(*n* = 555)
Absenteeism	0.05	0.05	0.04	0.01	0.01
Presenteeism	0.59	0.61	0.57	0.44	0.17
Total productivity loss	0.64	0.66	0.61	0.45	0.18

The majority (82.6 %) of employees were blue-collar (paint-house workers, press-metal workers, welders, assemblers, etc.); 17.4 % were white-collar (office workers, including managerial staff). Headache frequency in each was similar, but more blue-collar employees (3.7 %) than white (1.7 %) had headache on ≥15 days/month (Pearson *Χ*^2^ = 10.135; *P* = 0.038). Mean absenteeism rate was higher in blue-collar workers (0.04 ± 0.41 days/month) than white (0.02 ± 0.21; *P* = 0.033 [Student’s t-test]), while mean presenteeism rates were similar (0.52 ± 1.80 and 0.48 ± 1.53 days/month; *P* = 0.457).

### Interventional study

We ran the clinic for 15 months, during which, of the 2,688 employees who reported headache, 344 (12.8 %) requested and were given appointments. Of these, 211 (7.8 %) consulted and 133 (4.9 %) failed to attend (Table 3). Attendance was related to headache frequency, increasing from 2.9 % of those with one headache in the preceding month to 25.7 % of those with headache on 10–14 days/month (Table 3) (Pearson correlation: 0.200 [*P* <0.0005]).

**Table 3:** Employees requesting and attending appointments with the headache clinic according to headache frequency

Headache frequency (days/month)	Employees with headache (*N* = 2,688)	Employees requesting and receiving appointments (*n* = 344)	
		Employees consulting (*n* = 211)	Employees failing to attend (*n* = 133)
0–1	627	18 (2.9 %)	4 (0.6 %)
2–4	1,385	76 (5.5 %)	52 (3.8 %)
5–9	438	59 (13.5 %)	48 (11.0 %)
10–14	148	38 (25.7 %)	13 (8.8 %)
≥15	90	20 (22.2 %)	16 (17.8 %)

Of the 211 employees who came to the headache clinic, 171 were blue-collar (3.5 % of the participating blue-collar workforce) and 40 were white-collar (3.9 % of the participating white-collar workforce). Of the 133 employees who failed to attend, 111 were blue-collar (2.3 %) and 22 were white-collar (2.2 %). These differences were not significant.

#### Outcomes

Of the 211 who consulted for first appointments, only 73 (34.6 %) returned for follow-up appointments, and few of these did so at the times prescribed, which were appropriate for their optimum management. This greatly hindered their management. It also meant that outcome measurements were insufficient for useful analysis of outcomes. Therefore we have not reported them.

## Discussion

This year-long observational study confirmed, in a young mostly male workforce, the high productivity losses attributable to headache—by far the greater part being due to presenteeism rather than absenteeism. Also, a disproportionately large part was due to a small minority of employees with high-frequency headache. The findings are very much in agreement with those of our earlier study, which employed two different survey methods [9], but are preferable because they eliminate any seasonal effect. Employees were offered free health care on site in an attempt to show that productivity losses were at least in part recoverable. The attempt failed, because this apparently highly disabled workforce took little advantage of the offer.

In our methods, we diverged from our earlier study [9] by using HALT-30 rather than the standard HALT-90 Index. The latter counts days affected by headache during the preceding 3 months [13]. This balances the need in a *therapeutic* encounter with an individual patient to reflect his or her illness over a period of time against the problems of recall error when that period is prolonged. In a population measure, with a large sample, there was no need to reflect the states of individuals, whereas a measure over a shorter period of 1 month was likely to be more reliable through better recall. We assumed, in the total population, that annual lost time was correctly estimated from the sum of individual HALT-30 scores x 12.

In a workforce with a mean age of 32.5 years and 92.7 % male, we evaluated the effect of headache on productivity without classifying headache type. One-month prevalence of any headache was 45.4 %, very close to the 43.2 % we found previously [9]. While this estimate included migraine, TTH and all causes of headache occurring on ≥15 days/month, it cannot easily be related to estimates of 1-year prevalence [9] since a proportion of those with headache occurring less than once a month would have been excluded. Because of averaging, this did not impair the estimate of population burden, which we found in this young and mostly male workforce to be high, as we did previously [9]. Importantly, by carrying out a new year-long evaluation, we eliminated any influence of seasonal effect.

Lost productivity in the whole workforce was estimated at 6,452 hours/week, with a mean lost productive time per employee reporting any productivity loss (absenteeism and/or presenteeism) (*n* = 968) of 6.7 hours/week. There are few studies offering comparison [17–23] but, in one in the United States, Stewart et al. [17] reported mean productivity losses of 4.7 hours/week for migraine. In another [18], they investigated lost productive times due to each of a range of common pain conditions in the US workforce. The mean lost productive time for all headache types was 3.5 hours/week. These losses were lower than ours, which might be surprising since the US studies were population-based volunteer cohort studies selected to be representative of mixed workforces of the US population, while ours specifically targeted a rather homogenous group of young and mostly male employees working in a heavy-manufacturing industry in Turkey.

We do not wish to make too much of this. Comparisons with other countries with different cultures, dependent on studies using different methodologies and sampling methods and achieving lower participation rates, may not be of primary interest here. Furthermore, the possible reasons for high productivity losses in our particular workforce have been discussed before [9].

A consideration of more immediate relevance is the very high dependence of lost productivity on headache frequency (Table 1): while the group with headache on ≥15 days/month were 6.6 % (64/968) of the disabled group, they were responsible for a disproportionate 21.1 % of the productivity losses. The respective proportions for those with 5–14 headache days/month were 35.3 % (*n* = 342) and 45.9 %, while those with <5 headache days/month were 58.1 % (*n* = 562) of the disabled group and caused 58.1 % of the productivity losses. In other words, disproportionate productivity loss is seen with headache frequencies above once a week.

Yet the proportion of employees in our workforce with headache on ≥15 days/month (1.5 %) was low in comparison with findings of epidemiological studies [24, 25]. High-frequency headache does not sufficiently explain the high productivity losses in our workforce. Productivity losses were higher among female employees than males, as found elsewhere [2–6, 26], but, since our workforce was mostly male, this offers no explanation. As for the nature of the work (physically demanding for the majority of employees), although there was no difference in mean headache frequency between white- and blue-collar employees, headache on ≥15 days/month was more than twice as prevalent among the latter. Rate of absenteeism was two-fold higher in blue-collar employees while there was no difference in respect of presenteeism or overall lost productivity.

In summary, while productivity loss is clearly related to headache frequency, other not yet apparent explanations must be invoked for the high productivity losses in this workforce. It is worth noting that the absenteeism/presenteeism ratio was 1/16. Although again this was in complete concordance with our previous study [9], it is much exaggerated compared with the findings of other studies. We argued previously that this might be related to social and economic issues peculiar to the country. Incidentally, the ratio fell sharply as headache frequency increased above once a month, which may support this argument: absenteeism, being much more visible than presenteeism, is far less tolerated by employers. An entirely different explanation is also possible: as headache frequency increases, a coping mechanism is deployed.

Perhaps the more interesting points for discussion, in terms of their implications, arise from what we must regard as the failure of the interventional study. This apparently highly disabled workforce, suffering substantial productivity losses, were offered free health care on site and showed limited inclination to take advantage of it. Explanations are speculative, and will need testing. The first lies entirely with the employees: they had learnt to live with their headaches, they had developed coping mechanisms and they had low expectations for benefit from health care. Such attitudes, which erect a significant social barrier to health care, have been remarked upon before [27]. With this explanation, those using the service would have tended to be those worst affected, as happened (Table 3). The second is that, although the study was supported by senior management, employees were prevented (or at least discouraged) from taking time to attend by line-managers, to avoid local work disruptions. This would have appeared as a more random factor, affecting all severities equally, and this might be the case among those who made appointments but failed to attend (Table 3).

The implications for employers, who should have an interest in recovering lost productivity, are twofold. First, the focus of any intervention should be on those with frequent headache. Their numbers are relatively low but the impact of their headaches is very high. Second, it is not a sufficient intervention merely to make headache care available, although it is by no means clear what more needs to be done, or how to do it. The much wider implications are that these truisms can be extrapolated to the employed general public.

## Conclusions

We confirmed in the year-long observational study the high productivity losses in this young mostly male workforce, which correlated with but were not wholly explained by headache frequency. In a socioeconomic climate likely to discourage absenteeism, the absenteeism/presenteeism ratio was 1:16. As we found before, a small minority of employees with high-frequency headache contributed highly disproportionately to the productivity losses, and these should be the target of interventions aimed at productivity recovery. It is not clear what form such interventions should take. Our finding was that making headache care optimally available is, of itself, far short of a solution.
